# Network Meta-Analysis and Umbrella Review: Complementary, Not Competing, Tools of Evidence Synthesis

**DOI:** 10.7759/cureus.102123

**Published:** 2026-01-23

**Authors:** Abhijit Nair, Tuhin Mistry, Rakesh Garg

**Affiliations:** 1 Anesthesiology, Ibra Hospital, Ibra, OMN; 2 Anesthesiology, Ganga Medical Centre and Hospitals Pvt. Ltd., Coimbatore, IND; 3 Anesthesiology, All India Institute of Medical Sciences, New Delhi, IND

**Keywords:** evidence-base practice, network meta-analysis, qualitative evidence synthesis, systematic review and meta-analysis, umbrella review

## Abstract

The increasing volume of randomized trials and systematic reviews in medical and allied fields has led to greater reliance on advanced evidence synthesis methods to inform clinical practice and policy. Network meta-analysis (NMA) and umbrella reviews (URs) are frequently placed at the apex of evidence hierarchies and are sometimes perceived as competing methodologies; however, they address fundamentally different objectives. NMA operates at the trial level, integrating direct and indirect comparisons across a connected network to generate comparative effect estimates and, where appropriate, treatment rankings, making it particularly suited for focused clinical questions involving multiple active interventions. In contrast, URs function at the review level, synthesizing and critically appraising existing systematic reviews and meta-analyses to provide a panoramic overview of a broad topic, highlighting consistency, discordance, methodological limitations, and evidence gaps rather than producing new pooled estimates. Neither approach is inherently superior; their evidentiary value depends on the clinical question, the quality and structure of the available evidence, and the intended application. Used appropriately, NMAs provide decision-grade quantitative comparisons, while URs offer strategic orientation and research prioritization. Viewed together, they represent complementary, not competing, tools that strengthen evidence-informed decision-making in clinical practice.

## Editorial

Modern clinical practice increasingly depends on high-level evidence synthesis to guide practice. The growing volume of trials and systematic reviews in various medical and surgical specialties necessitates robust methods for integrating the data. Among these, network meta-analysis (NMA) and umbrella reviews (URs) are frequently placed at the apex of evidence synthesis, yet they serve distinct purposes. Understanding their respective roles helps clinicians and researchers interpret evidence accurately and apply it meaningfully to patient care.

What does a network meta-analysis do?

An NMA quantitatively compares more than two interventions by combining direct head-to-head evidence from randomized controlled trials with indirect evidence derived through a shared comparator within a connected network [1]. This framework allows estimation of relative treatment effects even when some interventions have not been directly compared. Beyond pooled effect estimates, NMAs frequently generate treatment rankings using P-scores or surface under the cumulative ranking curve (SUCRA) values, which are increasingly emphasized in clinical research [2]. While potentially informative, such rankings require cautious interpretation and must be contextualized by the precision of effect estimates, clinical plausibility, and methodological validity.

What does an umbrella review do?

A UR synthesizes published systematic reviews and/or meta-analyses (SRMAs) addressing a broad topic or intervention set [3,4]. It typically appraises the methodological quality of included SRMAs, maps overlap in primary studies, and summarizes where evidence converges or conflicts. By design, it does not re-pool primary trial data; instead, it integrates and critiques existing syntheses to offer a panoramic overview.

Essential differences and similarities

NMA operates at the trial level, yielding new quantitative estimates for specific clinical questions that are often suitable for use in guidelines or cost-effectiveness analyses. A UR operates at the review level and provides high-level orientation across interventions, populations, or outcomes, highlighting gaps and inconsistencies. Both approaches benefit from prospective protocol registration (e.g., PROSPERO) and transparent, reproducible methods [5]. However, their evidentiary contributions differ: NMAs refine comparative effectiveness, whereas URs contextualize the broader credibility and coherence of existing research. The fundamental distinctions between NMA and URs are summarized in Table 1.

**Table 1:** Comparison of NMA and UR SRMA: systematic review and/or meta-analysis; NMA: network meta-analysis; UR: umbrella review; AI: artificial intelligence

Aspect	NMA	UR
Unit of analysis	Individual randomized trials or observational studies	Published SRMAs
Primary purpose	Quantitatively compares multiple interventions within a connected evidence network	Synthesizes and appraises existing SRMAs across a broad topic
Evidence source	Direct and indirect trial data	Secondary analyses (SRMA)
Output	Effect estimates and ranking of competing interventions	Summary of strength, direction, and consistency of existing evidence
Scope of question	Focused, intervention-specific (e.g., optimal analgesic technique)	Broad, domain-level (e.g., analgesic strategies in peri-operative care)
Analytical approach	Bayesian or frequentist models integrating multiple comparisons	Narrative and tabular synthesis, sometimes quantitative re-analysis of SRMA data
Assumptions required	Transitivity, homogeneity, and consistency across trials	Minimal statistical assumptions, relies on quality and overlap of included SRMAs
Typical challenges	Network geometry gaps, inconsistent comparators, and small-study bias	Overlapping primary studies, variable SRMA quality, heterogeneity of results
AI integration (emerging)	Automated study identification, bias detection, and network optimization	Limited; potential for automated SRMA retrieval and overlap detection
Use in anesthesia and perioperative care	Comparative effectiveness of nerve blocks, adjuvants, and antiemetic regimens	Broad synthesis of perioperative outcomes, safety, and recovery domains
Ideal application	Guideline and decision-level quantitative comparisons	Strategic, policy-level evidence overview and research gap mapping

Assumptions and typical biases

Credible NMAs depend on several critical assumptions, including transitivity (comparability of effect modifiers across trials), homogeneity (consistency within direct comparisons), and consistency/coherence (agreement between direct and indirect evidence), alongside appropriate handling of multi-arm trials and small-study effects [6].

Failure to address these assumptions can substantially undermine the internal validity of NMAs, leading to distorted effect estimates, spurious treatment hierarchies, and false precision. Violations of transitivity may render indirect comparisons clinically incoherent; inconsistency between direct and indirect evidence may indicate structural flaws within the network; and unrecognized small-study effects or publication bias may exaggerate the apparent superiority of interventions [7]. Such distortions are particularly consequential in perioperative research, where heterogeneous populations, multimodal interventions, and composite outcomes are common. Because NMAs are vulnerable to biases arising from how evidence is assembled, analyzed, and interpreted, recently developed frameworks such as the Risk of Bias in NMA (RoB NMA) tool emphasize structured evaluation of network geometry, effect modifiers, and statistical synthesis to prevent misleading effect estimates and treatment rankings [8].

URs, by contrast, inherit the strengths and weaknesses of the SRMAs they include. Their validity depends on the methodological quality of those SRMAs (searches, risk-of-bias tools, synthesis methods), the extent of overlap in primary studies (risking double-counting), and the heterogeneity or discordance among pooled results [9]. Without careful appraisal, URs may amplify rather than resolve bias.

Quality appraisal and contextualization of reliability

The credibility of both NMAs and URs fundamentally depends on structured quality assessment. For URs in particular, A MeaSurement Tool to Assess Systematic Reviews 2 (AMSTAR-2) provides a validated framework to appraise the methodological rigor of included systematic reviews across 16 critical domains, including protocol registration, adequacy of the literature search, risk-of-bias assessment, handling of heterogeneity, and appropriateness of meta-analytic methods [4]. Importantly, rather than generating a numerical score, it offers an overall confidence rating (high, moderate, low, or critically low) in the results of each review. This aligns well with UR methodology, where the objective is to contextualize trustworthiness rather than to generate new pooled estimates. For NMAs, structured risk-of-bias assessment of included trials, evaluation of publication bias, and appraisal of certainty of evidence using adapted Grading of Recommendations Assessment, Development, and Evaluation (GRADE) tools for NMA or Confidence in Network Meta-Analysis (CINeMA) frameworks are essential to contextualize network findings [10]. Without such systematic appraisal, higher-order syntheses risk conferring unwarranted authority on methodologically fragile evidence.

Artificial intelligence and software tools in network meta-analysis and umbrella review workflows

Artificial intelligence (AI) and machine-learning tools are increasingly being explored to enhance evidence synthesis workflows by streamlining key tasks such as literature search, screening, data extraction, and methodological quality assessment. Fine-tuned large language models (LLMs) have demonstrated potential to support structured data extraction and methodological appraisal of systematic reviews, improving efficiency and consistency compared with fully manual processes [11]. AI-enabled screening algorithms and active-learning platforms can substantially reduce reviewer workload and accelerate evidence synthesis, although human oversight remains essential to preserve methodological rigor.

Beyond these supportive roles, recent feasibility studies have extended AI applications directly into NMA workflows. Early work using Generative Pre-trained Transformer (GPT-4) demonstrated the ability to perform structured data extraction, generate executable R scripts for Bayesian NMAs, and provide preliminary interpretation of forest plots and heterogeneity outputs, albeit with the need for careful technical verification [12]. More recent “AI-statistician” pipelines using LLMs have further automated model selection, execution, convergence diagnostics, and interpretation, with validation against established National Institute for Health and Care and Excellence (NICE) technical support document examples [13]. These developments suggest a future in which parts of NMA production may become semi-automated, supporting living and rapidly updatable evidence syntheses.

Alongside conceptual advances, a growing ecosystem of software platforms now supports practical workflows in NMA and URs. Machine-learning-assisted screening and review-management systems such as Rayyan, Covidence, and Elicit are widely used to facilitate title/abstract and full-text screening, support independent reviewer workflows, and assist early data extraction [14]. Citation-analysis and reference-management tools incorporating AI functions, including Scite and EndNote, are increasingly employed to support de-duplication, citation mapping, and management of large evidence libraries [15].

For URs, dedicated platforms such as MetaUmbrella have been developed to support evidence mapping, assessment of credibility, management of overlapping primary studies, and application of evidence-grading frameworks [16]. In the context of NMA, web-based analytical platforms such as NetMetaEasy facilitate implementation of frequentist NMA through structured interfaces, while emerging LLM-based workflows and AI-driven analytical environments, including GPT-4-based platforms such as Meta-MAR, have demonstrated feasibility for structured data extraction, generation of statistical code, execution of network models, and preliminary interpretation of network outputs [17,18]. Together, these tools illustrate a shift toward semi-automated, human-supervised evidence-synthesis pipelines.

Nevertheless, important challenges remain, including algorithmic bias, limited transparency of decision pathways, difficulty in operationalizing methodological constructs such as transitivity, inconsistency, or overlap, and the risk of propagating errors at scale. Consequently, AI should currently be regarded as a methodological adjunct rather than a substitute for protocol-driven evidence synthesis, structured quality appraisal, and expert clinical interpretation.

Which is superior in the evidence hierarchy?

Traditional pyramids often place SRMAs at the top. Some argue URs sit even higher because they synthesize across multiple SRMAs [19]. In practice, hierarchy is not absolute but question-dependent. A well-conducted NMA based on high-quality trials may deliver more actionable estimates and rankings for a precise decision than a UR that summarizes prior analyses without new pooling. Conversely, when the aim is strategic oversight, to contrast results across reviews, expose gaps, or harmonize messages for broad policy, a UR can be more informative than any single NMA [20]. Quality, scope, and coherence, not labels, should determine evidentiary weight.

When to choose which?

Select an NMA when multiple active comparators exist, trials form a connected network, and the decision requires comparative effectiveness estimates or rankings (e.g., choosing among several analgesic adjuvants). Ensure a careful assessment of transitivity and consistency, pre-specify effect modifiers, and use models that are robust to small-study and multi-arm issues [1,2,6]. Select a UR when stakeholders need an integrated map of what multiple SRMAs conclude about a broad domain (e.g., perioperative analgesic strategies across surgical populations), including where evidence aligns, conflicts, or remains uncertain. Prioritize rigorous SRMA appraisal, overlap mapping, and transparent grading of certainty [9,19,20].

Decision implications

The NMA outputs (relative effects, ranks, and SUCRA curve) can inform decision models, guideline panels, and cost-effectiveness analyses, provided the assumptions hold [1,2,6]. URs offer contextual clarity: they collate signals, expose redundancy, and direct future primary research or de-novo meta-analysis where evidence is thin or inconsistent [8-11]. Both approaches benefit from living-update frameworks as evidence evolves.

In anesthesia and perioperative medicine, both approaches are increasingly applied to synthesize evidence across regional analgesic techniques, airway interventions, and multimodal perioperative pharmacological strategies. High-quality NMAs have informed clinical guidelines, for example, comparing peripheral nerve block approaches, adjuvants, or antiemetic regimens, while URs have summarized broader perioperative safety and recovery domains. Integrating these frameworks can refine guideline development and strengthen evidence-informed practice in anesthesia.

A pragmatic synthesis

Rather than viewing one as superior to the other, it is more constructive to regard them as complementary: NMA delivers decision-grade estimates within a focused question, while URs provide decision-support at the landscape level. Where feasible, triangulating insights, using URs to scope and prioritize, and NMA to quantify, can align evidence synthesis with real-world clinical and policy needs.

To conclude, NMAs and URs occupy different, yet synergistic, niches in clinical research. Rather than representing competing hierarchies, they should be viewed as complementary tools whose value depends on methodological rigor, clinical context, and critical interpretation. Emphasizing structured appraisal and conceptual clarity over hierarchy alone may help clinicians better translate high-level evidence into safe, effective, and context-appropriate patient care.
